# Safety and Immunogenicity of a Fourth Dose of Omicron BA.1–Adapted BNT162b2 COVID-19 Vaccines in Adults 18–55 Years Old

**DOI:** 10.1093/cid/ciag026

**Published:** 2026-01-21

**Authors:** Patricia Winokur, Oyeniyi Diya, David Fitz-Patrick, Michael Dever, Juleen Gayed, Stephen Lockhart, Xia Xu, Ying Zhang, Vishva Bangad, L Tyler Wadsworth, Kevin Cannon, Jose F Cardona, Lisa Usdan, John Ginis, Federico J Mensa, Jing Zou, Xuping Xie, Claire Lu, Sandra Buitrago, Ingrid L Scully, David Cooper, Kenneth Koury, Kathrin U Jansen, Özlem Türeci, Uğur Şahin, Kena A Swanson, William C Gruber, Nicholas Kitchin

**Affiliations:** Division of Infectious Diseases, Carver College of Medicine, University of Iowa, Iowa City, Iowa, USA; Vaccine Research and Development, Pfizer Ltd, Hurley, United Kingdom; East-West Medical Research Institute, Honolulu, Hawaii, USA; Clinical Neuroscience Solutions, Orlando, Florida, USA; Vaccine Research and Development, Pfizer Ltd, Hurley, United Kingdom; Vaccine Research and Development, Pfizer Ltd, Hurley, United Kingdom; Vaccine Research and Development, Pfizer Inc, Collegeville, Pennsylvania, USA; Vaccine Research and Development, Pfizer Inc, Pearl River, NewYork, USA; Vaccine Research and Development, Pfizer Inc, Collegeville, Pennsylvania, USA; Sundance Clinical Research, St Louis, Missouri, USA; PMG Research of Wilmington, Wilmington, North Carolina, USA; Indago Research & Health Center, Inc, Hialeah, Florida, USA; CNS Healthcare, Memphis, Tennessee, USA; Vaccine Research and Development, Pfizer Inc, Collegeville, Pennsylvania, USA; BioNTech, Mainz, Germany; Department of Biochemistry and Molecular Biology, University of Texas Medical Branch, Galveston, Texas, USA; Department of Biochemistry and Molecular Biology, University of Texas Medical Branch, Galveston, Texas, USA; Vaccine Research and Development, Pfizer Inc, Pearl River, NewYork, USA; Vaccine Research and Development, Pfizer Inc, Pearl River, NewYork, USA; Vaccine Research and Development, Pfizer Inc, Pearl River, NewYork, USA; Vaccine Research and Development, Pfizer Inc, Pearl River, NewYork, USA; Vaccine Research and Development, Pfizer Inc, Pearl River, NewYork, USA; Vaccine Research and Development, Pfizer Inc, Pearl River, NewYork, USA; BioNTech, Mainz, Germany; BioNTech, Mainz, Germany; Vaccine Research and Development, Pfizer Inc, Pearl River, NewYork, USA; Vaccine Research and Development, Pfizer Inc, Pearl River, NewYork, USA; Vaccine Research and Development, Pfizer Ltd, Hurley, United Kingdom

**Keywords:** BNT162b2, COVID-19, SARS-CoV-2, Omicron, BA.1

## Abstract

**Background:**

Emergence of severe acute respiratory syndrome coronavirus 2 sublineages warrants the use of sequence-adapted vaccines to provide protection against coronavirus disease 2019.

**Methods:**

In this phase 3 trial, adults 18‒55 years old who had previously received three 30 μg doses of BNT162b2 vaccine were randomized to receive a 60 or 30 μg dose of bivalent Omicron BA.1‒adapted BNT162b2 comprising equal amounts of ancestral and monovalent messenger RNA BA.1 (bivalent BA.1) or a 60 μg dose of monovalent Omicron BA.1‒adapted BNT162b2 (monovalent BA.1). Safety (local reactions, systemic events, adverse events [AEs], and serious AEs) was the primary objective. Exploratory analyses assessed immune responses against Omicron BA.1, BA.4, and BA.5 subvariants and ancestral strain.

**Results:**

Among the 1054 randomized participants who received monovalent BA.1 or bivalent BA.1, frequencies of local reactions, systemic events, and AEs were slightly higher with 60 µg monovalent BA.1 than either bivalent BA.1 dose level. One month after vaccination, bivalent BA.1 (30 and 60 μg) and monovalent BA.1 (60 μg) induced substantial neutralizing responses against Omicron BA.1 (50% neutralizing titer geometric mean fold rises [GMFRs], 15.4 [95% CI 12.4–19.2], 17.1 [13.7–21.4], and 24.6 [19.3–31.4], respectively) and ancestral strain (GMFRs, 6.2 [5.1–7.6], 7.3 [6.0–8.9], and 7.0 [5.7–8.7], respectively). In a smaller (n = 30/treatment arm) sentinel cohort, all study vaccines modestly neutralized Omicron BA.4 and BA.5.

**Conclusions:**

Bivalent and monovalent BA.1‒adapted vaccines had a safety profile similar to the original BNT162b2 30 μg and induced substantial neutralizing responses against Omicron BA.1 and ancestral strains.

**Clinical Trials Registration:**

NCT04955626.

## Teaser

Bivalent and monovalent BA.1‒adapted COVID-19 vaccines had a safety profile similar to BNT162b2 (30 μg), induced substantial neutralizing responses against Omicron BA.1 and ancestral strains, and neutralized BA.4 and BA.5 sublineages to a lesser extent in adults 18‒55 years old.

Vaccination remains an important intervention for coronavirus disease 2019 (COVID-19) prevention globally [1]. BNT162b2, a messenger RNA (mRNA) vaccine encoding the SARS-CoV-2 spike protein [2], demonstrated 95%‒100% efficacy after the two 30 μg dose primary series [3, 4]. Following these phase 2‒3 results obtained early during the pandemic, the primary series with or without a third dose of original BNT162b2 provided broad protection against various severe acute respiratory syndrome coronavirus 2 (SARS-CoV-2) variants of concern (VOC) with relatively low potential to escape vaccine-elicited immunity [5–9]. However, compared with previous VOC, original BNT162b2 and other COVID-19 vaccines demonstrated reduced effectiveness against the Omicron variant [10]. Immunity waning has also been more pronounced for Omicron sublineages relative to previous variants [11–13] and less antigenically distant versus ancestral strains (eg, Beta and Delta) [14].

Variant-adapted vaccines, as encouraged by regulatory authorities [15, 16], improved effectiveness against newer lineages [17]. To explore safety and immunogenicity of various BNT162b2 boosting strategies, a master phase 3 study (NCT04955626) comprising several substudies was developed [18, 19]. One substudy evaluated the safety and immunogenicity of a fourth vaccination with monovalent Omicron BA.1‒adapted BNT162b2 (monovalent BA.1) or bivalent Omicron BA.1‒adapted BNT162b2 (bivalent BA.1) in adults >55 years old after 3 doses of original BNT162b2 30 µg. Compared with BNT162b2, the monovalent BA.1 vaccine differs from the original BNT162b2 (encoding SARS-CoV-2 ancestral strain) at amino acid substitutions specific to the Omicron BA.1 spike. The bivalent vaccine contains equal amounts of ancestral and Omicron BA.1 spike mRNA coformulated into lipid nanoparticles [19]. With the emergence of Omicron BA.1 and BA.4/BA.5 (designated together as they have the same spike sequence) during the study, the protocol was adapted to evaluate Omicron BA.1‒adapted vaccines neutralization of BA.4/BA.5 sublineages [19, 20]. Among 1846 individuals >55 years old, boosting with monovalent or bivalent Omicron BA.1‒adapted vaccines had a safety profile similar to the original BNT162b2 30 μg and induced greater neutralizing responses against ancestral and Omicron BA.1, BA.4, and BA.5 variants than a fourth dose of BNT162b2 30 µg [19]. Here, we present safety and immunogenicity of a fourth dose of Omicron BA.1–adapted BNT162b2 vaccines in individuals 18‒55 years old with a mean follow-up of 6.4 months.

## METHODS

### Study Design and Participants

This randomized, observer-blinded phase 3 study [18, 19] evaluated safety and immunogenicity of monovalent and bivalent boosting strategies with a BA.1‒adapted BNT162b2 vaccine. Study visits were conducted between 22 February 2022 and 17 January 2023. Participants were randomized to bivalent BA.1 60 μg (30 μg BNT162b2-Omi.BA.1 + 30 μg BNT162b2), bivalent BA.1 30 μg (15 μg BNT162b2-Omi.BA.1 + 15 μg BNT162b2), or monovalent BA.1 60 μg (60 μg BNT162b2-Omi.BA.1). Healthy individuals 18‒55 years old who previously received 3 doses of BNT162b2 30 µg, with the last dose 5‒12 months before randomization, were eligible. Key exclusion criteria included immunocompromised individuals, previous clinical or microbiologic diagnosis of COVID-19, and receipt of other medications or vaccines intended to prevent COVID-19.

The protocol, amendments, and other relevant study documents were reviewed and approved by an Institutional Review Board/Independent Ethics Committee. The study was conducted in accordance with the principles of the Declaration of Helsinki Council, Council for International Organizations of Medical Sciences International Ethical Guidelines, applicable International Council of Harmonisation Good Clinical Practice Guidelines, and all other applicable laws and regulations. All participants provided written informed consent.

### Procedures

Initially, a sentinel cohort of 90 participants was enrolled, with 30 participants each randomized to receive either a 60 or 30 µg dose of bivalent BA.1 or a 60 µg dose of monovalent BA.1. The sentinel cohort was used to evaluate reactogenicity of the higher vaccine doses before expanding to a larger cohort. An independent review committee confirmed that safety data through Day 7 from this cohort (see Supplementary Data) were acceptable before enrolling the expanded cohort, which comprised an additional 900 participants randomized 3:1:2 to bivalent BA.1 60 µg, bivalent BA.1 30 µg, or monovalent BA.1 60 µg. Randomization was conducted using an interactive response technology system, with preparation and administration of study interventions carried out by study personnel unblinded to vaccine allocation. All other study personnel, including the investigator, investigator staff, and participants, were blinded.

Details regarding vaccine formulation and administration are in the Supplementary Data.

### Objectives, Endpoints, and Assessments

Primary safety endpoints included percentages of participants reporting local reactions and systemic events through 7 days after vaccination and adverse events (AEs) and serious AEs (SAEs) through 1 and 6 months after vaccination. Adverse events and SAEs were actively collected from the time the participant provides informed consent through 1 month after the participant's study vaccination and through 6 months for SAEs; any AEs occurring up to 48 hours after any subsequent blood draw were recorded. Participants recorded information regarding local reactions, systemic events, and antipyretic medication use in an electronic diary; study investigators actively collected information regarding AEs and SAEs from participants.

Characterization of immune responses with Omicron BA.1–adapted vaccines among the 18‒55-year-old cohort was an exploratory objective. For the sentinel cohort, a fluorescence focus reduction neutralization test assay evaluated SARS-CoV-2 serum neutralization titers against ancestral strain, BA.1, and BA.4/BA.5 [21–24]. For the expanded cohort, SARS-CoV-2 50% neutralization titers against ancestral strain and BA.1 were tested using a validated microneutralization assay. Assays for Omicron BA.1 used a recombinant virus with the Omicron variant full spike gene on the genetic background of ancestral strain (USA-WA1/2020). Immunogenicity endpoints included geometric mean titers (GMTs) at each timepoint, geometric mean fold rises (GMFRs) from baseline to each postbaseline timepoint, and percentages of participants with seroresponse (ie, ≥4-fold rise from baseline or ≥4× lower limit of quantitation [LLOQ] for baseline measurements < LLOQ) before vaccination and at each subsequent timepoint. Geometric mean ratios (GMRs) and differences in percentages of participants with seroresponse between vaccine groups were calculated in supportive analyses. Confirmed COVID-19 and severe COVID-19 cases that developed during the study are also described (see Supplementary Data).

### Statistical Analysis

Sample sizes for each group were determined based on consideration of an acceptable safety database. The safety population included all participants who received study intervention. Descriptive statistics are provided for safety endpoints, including percentages of participants with the indicated endpoint and associated Clopper‒Pearson 95% CIs. Local reactions and systemic events were graded as mild, moderate, severe, or potentially life-threatening (Supplementary Table 1). Adverse events were categorized using Medical Dictionary for Regulatory Activities (MedDRA) terms (version 25.1).

A subset of participants was included in the immunogenicity assessment. This subset comprised approximately 150 participants 18‒55 years old randomized to each vaccine group, with the youngest 150 participants from the >55-year-old group who received the 30 µg dose of bivalent BA.1 as a reference group. Immunogenicity analyses were based on the evaluable immunogenicity population, which included all eligible participants with or without previous evidence of SARS-CoV-2 infection up to 1 month after vaccination who received study intervention to which they were randomized, had a valid and determinate immunogenicity result collected within 28‒42 days after vaccination, and no major protocol deviations. Details regarding individuals without evidence of SARS-CoV-2 infection through 1 month after vaccination are in the Supplementary Data.

Geometric mean titers were calculated by exponentiating the mean of logarithmically transformed assay results and corresponding 2-sided 95% CIs based on the Student *t* distribution. Geometric mean fold rises and GMRs were calculated by exponentiating the mean of the difference of logarithmically transformed assay results (later timepoint minus earlier timepoint [GMFRs] or between vaccine groups [GMRs]) and corresponding 2-sided 95% CIs based on the Student *t* distribution. Percentages and associated Clopper‒Pearson 95% CIs were calculated for seroresponses, and Miettinen‒Nurminen 95% CIs were calculated for differences in seroresponses.

## RESULTS

### Participants

**Figure 1.**
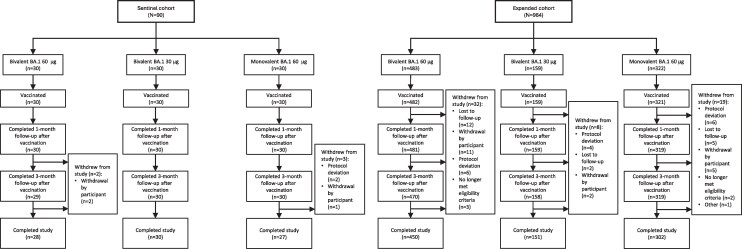
Randomization and administration of a fourth vaccine dose after having previously received three 30 μg doses of the BNT162b2 vaccine for both the sentinel (left) and expanded (right) cohorts. *A*, Protocol deviations could include receipt of disallowed medication or nonstudy vaccines, or not meeting inclusion/exclusion criteria. *B*, The reason for withdrawal for 1 participant was classified as “Other.” Participants were recruited from 46 sites in the United States.

Of 1054 participants randomized from 40 sites in the United States (sentinel cohort, n = 90; expanded cohort, n = 964; Figure 1), 1052 were vaccinated (bivalent BA.1 60 µg, n = 512; bivalent BA.1 30 µg, n = 189; monovalent BA.1 60 µg, n = 351). In the expanded cohort, 962 of 964 participants were vaccinated, and 959 completed the 1-month postvaccination visit by the cutoff date of 25 August 2022 and were included in the analyses for immunogenicity, local reaction, and systemic events. The median follow-up duration after vaccination for these endpoints was 2.4 months. Overall, 903 participants completed the 6-month postvaccination visit and were included in the safety analysis. The mean follow-up duration was 6.3 months.

Patient demographics were similar between groups (Table 1). Median age at vaccination was 42 years, 83.3% of participants were White, 6.7% were Black, and 11.1% were Hispanic/Latino. Median time from BNT162b2 vaccination 3 to study vaccination was 8.7 months. Demographics for the sentinel cohort are in Supplementary Table 4.

**Table 1.** Patient Demographics and Baseline Clinical Characteristics (Expanded Cohort Safety Population)

Characteristic	Expanded Cohort (n = 962)		
	Bivalent BA.1 60 µg (n = 482)	Bivalent BA.1 30 µg (n = 159)	Monovalent BA.1 60 µg (n = 321)
Sex, n (%)			
Male	213 (44.2)	70 (44.0)	141 (43.9)
Female	269 (55.8)	89 (56.0)	180 (56.1)
Age (range) at vaccination, median, years	41.0 (18–55)	43.0 (19–55)	42.0 (18–55)
Race, n (%)			
White	389 (80.7)	124 (78.0)	250 (77.9)
Black	34 (7.1)	10 (6.3)	32 (10.0)
Asian	42 (8.7)	20 (12.6)	25 (7.8)
Other^a^	16 (3.3)	5 (3.1)	12 (3.7)
Not reported	1 (0.2)	0	2 (0.6)
Ethnicity, n (%)			
Hispanic/Latino	66 (13.7)	17 (10.7)	39 (12.1)
Non-Hispanic/non-Latino	413 (85.7)	142 (89.3)	282 (87.9)
Not reported	3 (0.6)	0	0
Baseline SARS-CoV-2 status, n (%)			
Positive^b^	119 (24.7)	35 (22.0)	90 (28.0)
Positive NAAT	11 (2.3)	3 (1.9)	7 (2.2)
Negative^c^	363 (75.3)	124 (78.0)	230 (71.7)
Missing	0	0	1 (0.3)
Median time since prior receipt of BNT162b2 dose 3 (range), months	8.7 (5.4–12.3)	8.7 (5.6–13.0)	8.7 (5.1–12.3)
Time since prior receipt of BNT162b2 Dose 3, months, n (%)			
<5	0	0	0
≥5 to <7	29 (6.0)	16 (10.1)	39 (12.1)
≥7 to <9	272 (56.4)	83 (52.2)	150 (46.7)
≥9 to <11	92 (19.1)	31 (19.5)	75 (23.4)
≥11 to <12	61 (12.7)	19 (11.9)	41 (12.8)
>12	28 (5.8)	10 (6.3)	16 (5.0)
Body mass index, n (%)			
Underweight (<18.5 kg/m^2^)	8 (1.7)	3 (1.9)	4 (1.2)
Normal weight (≥18.5–24.9 kg/m^2^)	145 (30.1)	40 (25.2)	87 (27.1)
Overweight (≥25.0–29.9 kg/m^2^)	133 (27.6)	41 (25.8)	95 (29.6)
Obese (≥30.0 kg/m^2^)	196 (40.7)	75 (47.2)	135 (42.1)

Abbreviations: COVID-19, coronavirus disease 2019; N-binding, SARS-CoV-2 nucleoprotein–binding; NAAT, nucleic acid amplification test; SARS-CoV-2, severe acute respiratory syndrome coronavirus 2.

^a^Includes American Indian or Alaska Native, Native Hawaiian or other Pacific Islander, and multiracial.

^b^Positive N-binding antibody result at baseline, positive NAAT result at baseline, or medical history of COVID-19.

^c^Negative N-binding antibody result at baseline, negative NAAT result at baseline, and no medical history of COVID-19.

### Safety

**Figure 2.**
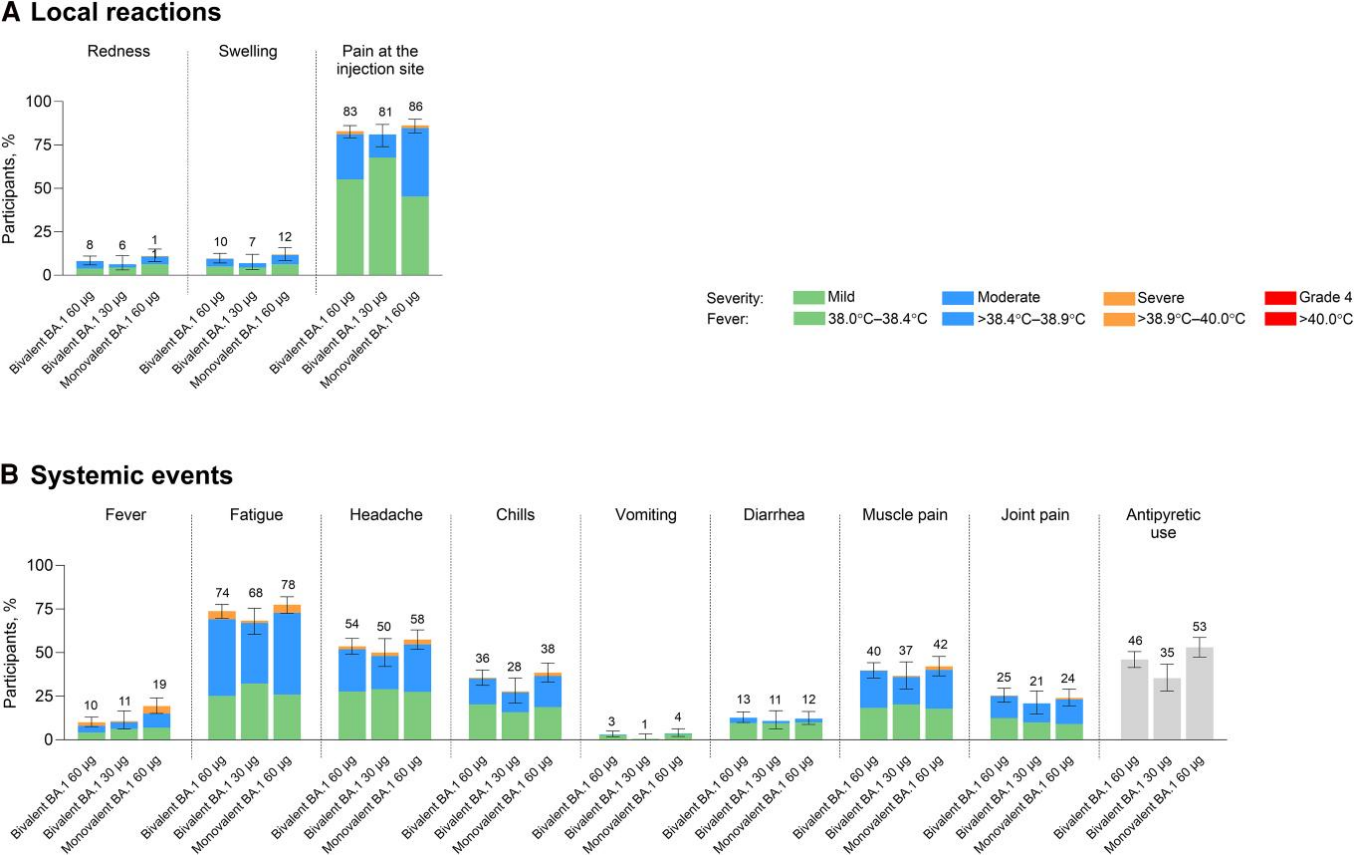
Local reactions (*A*) and systemic events (*B*) reported within 7 d of vaccination in the expanded cohort (safety population). Data are presented for the safety population (all participants who received the study intervention) in the expanded cohort. Severity grading of the specific local reactions and systemic events is provided in Supplementary Table 1. Bars represent the 95% CIs, and numbers above the bars indicate the percentage of participants in each group reporting any severity for the specified event. For antipyretic use, bars represent the percentage of participants who used antipyretics and are therefore not color coded. The bivalent BA.1 60 μg group included participants who received 30 μg BNT162b2-Omi.BA.1 + 30 μg BNT162b (n = 480), the bivalent BA.1 30 μg group included those who received 15 μg BNT162b2-Omi.BA.1 + 15 μg BNT162b2 (n = 158), and the monovalent BA.1 60 μg included those who received 60 μg BNT162b2-Omi.BA.1 (n = 320). Abbreviation: CI, confidence interval.

In the expanded cohort safety population, frequencies of local reactions and systemic events were generally higher with bivalent and monovalent BA.1 60 µg than bivalent BA.1 30 µg (Figure 2). Most local reactions were mild to moderate, with injection site pain being the most common. Median time to onset across groups was 1‒2 days, and all events resolved within a median of 1‒3 days. Severe local reactions with bivalent BA.1 60 µg and monovalent BA.1 60 µg included injection site pain (≤1.7%), redness (≤0.3%), and swelling (≤0.3%); no severe local reactions with bivalent BA.1 30 µg were reported.

Most systemic events were mild to moderate, with fatigue being the most common. Fever was more common with monovalent BA.1 60 µg (19.4%) than bivalent 60 or 30 µg (10.0%‒10.8%). Other systemic events were generally similar between groups. Median onset time was typically 2 days, and all events resolved within a median of 1‒2 days. Overall severe systemic events included fatigue (≤4.7%), headache (≤2.8%), new/worsened muscle pain (≤2.2%), chills (≤1.9%), and new/worsened joint pain (≤0.9%).

Through 1 month after study vaccination, the percentage of participants in the monovalent BA.1 60 µg group reporting AEs (9.3%) was slightly higher than the bivalent BA.1 groups (60 µg, 7.7%; 30 µg, 6.3%) (Table 2). Through 6 months after vaccination, the percentage of participants in the monovalent BA.1 60 µg group reporting AEs (12.1%) remained slightly higher than the bivalent BA.1 groups (60 µg, 10.6%; 30 µg, 9.4%). Many AEs were consistent with reactogenicity events (eg, injection site pain, diarrhea, and pyrexia). Vaccine-related AEs were uncommon (≤5.3% overall), as were severe AEs (≤1.2%) and SAEs (≤0.1.3%). No cases of myocarditis/pericarditis, Bell's palsy (or facial paralysis/paresis), or vaccine-related anaphylaxis were reported. One participant reported a life-threatening AE of perforated appendicitis 82 days after receipt of bivalent 60 μg, which was considered by the investigator to not be related to study vaccine. No withdrawals due to AEs or deaths were reported. AEs for the sentinel cohort through 6 months after vaccination are described in the Supplementary Results and summarized in Supplementary Table 5.

**Table 2.** Summary of AEs Through 1 Month and 6 Months After Study Vaccination (Expanded Cohort Safety Population)

Adverse Event	1 Month After Vaccination			6 Month After Vaccination		
	Bivalent BA.1 60 µg (n = 482)	Bivalent BA.1 30 µg (n = 159)	Monovalent BA.1 60 µg (n = 321)	Bivalent BA.1 60 µg (n = 482)	Bivalent BA.1 30 µg (n = 159)	Monovalent BA.1 60 µg (n = 321)
Any AE, n (%)	37 (7.7)	10 (6.3)	30 (9.3)	51 (10.6)	15 (9.4)	39 (12.1)
Related	17 (3.5)	3 (1.9)	14 (4.4)	21 (4.4)	4 (2.5)	17 (5.3)
Severe	2 (0.4)	0	3 (0.9)	4 (0.8)	1 (0.6)	4 (1.2)
Life-threatening	1 (0.2)	0	0	2 (0.4)	0	0
Any SAE, n (%)	0	0	2 (0.6)	3 (0.6)	2 (1.3)	3 (0.9)
Related	0	0	0	0	0	0
Severe	0	0	2 (0.6)	2 (0.4)	1 (0.6)	3 (0.9)
Life-threatening	0	0	0	1 (0.2)	0	0
Any nonserious AE, n (%)	37 (7.7)	10 (6.3)	30 (9.3)	50 (10.4)	14 (8.8)	38 (11.8)
Related	17 (3.5)	3 (1.9)	14 (4.4)	21 (4.4)	4 (2.5)	17 (5.3)
Severe	2 (0.4)	0	2 (0.6)	2 (0.4)	0	2 (0.6)
Life-threatening	1 (0.2)	0	0	1 (0.2)	0	0
Any AE leading to withdrawal, n (%)	0	0	0	0	0	0
Death, n (%)	0	0	0	0	0	0

Abbreviations: AEs, adverse events; SAEs, serious adverse events.

### Immunogenicity

The evaluable immunogenicity population comprised 444 participants in the immunogenicity subset of the expanded cohort (bivalent BA.1 60 µg, n = 148; bivalent BA.1 30 µg, n = 152; monovalent BA.1 60 µg, n = 144). Among those, 300 had no evidence of SARS-CoV-2 infection up to 1 month after vaccination (bivalent BA.1 60 µg, n = 104; bivalent BA.1 30 µg, n = 105; monovalent BA.1 60 µg, n = 91).

#### Omicron BA.1 Variant

**Figure 3.**
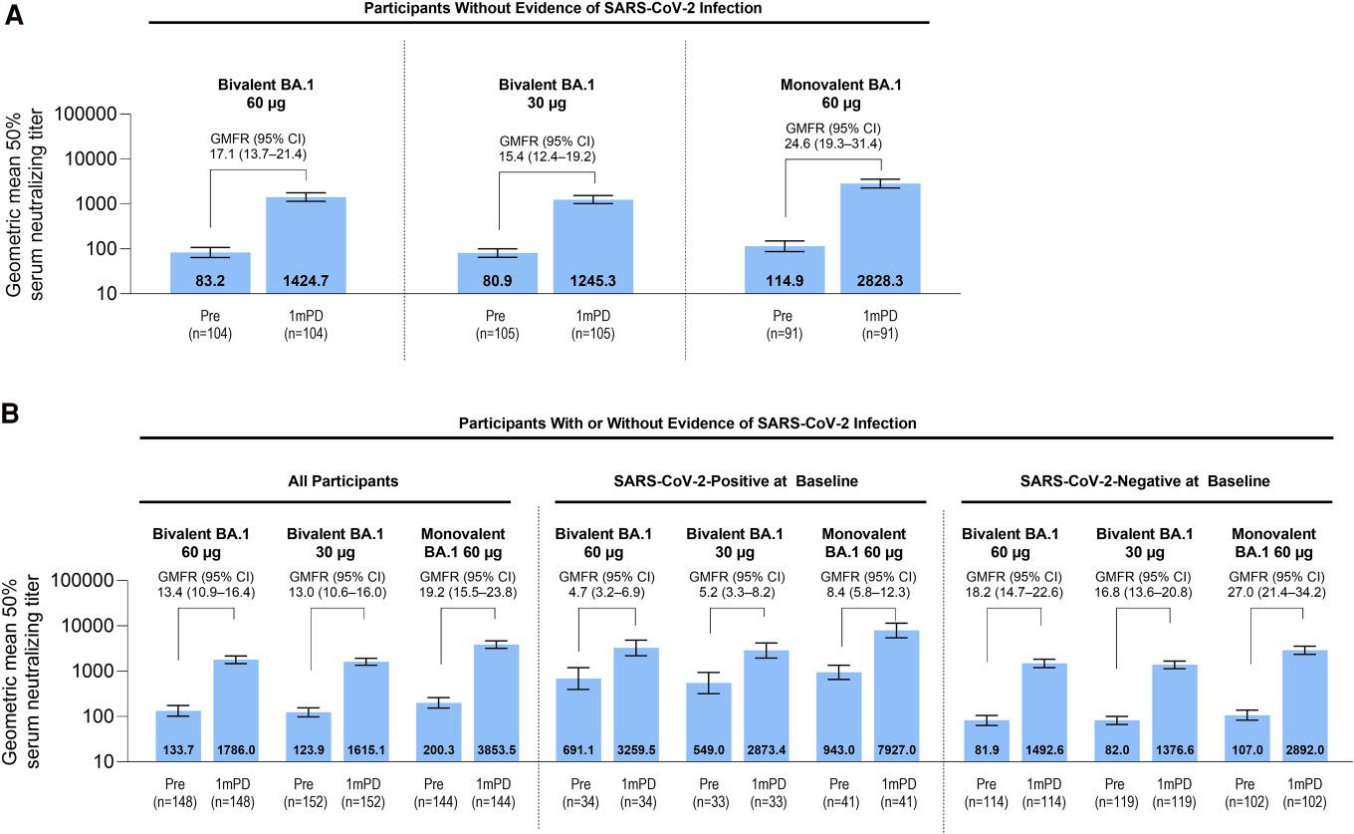
SARS-CoV-2 50% neutralization GMTs (95% CI) and GMFRs (95% CI) before and at 1 m after vaccination for the Omicron BA.1 strain in expanded cohort participants without evidence of SARS-CoV-2 infection (*A*), those with or without evidence of SARS-CoV-2 infection and by baseline SARS-CoV-2 status (*B*) (evaluable immunogenicity population). For GMTs and GMFRs, assay results below the LLOQ were set to 0.5 × LLOQ in the analysis. Abbreviations: 1mPD, 1 m postdose; CI, confidence interval; GMFR, geometric mean fold rise; GMT, geometric mean titer; LLOQ, lower limit of quantitation; NT50, 50% neutralizing titer; pre, before vaccination; SARS-CoV-2, severe acute respiratory symdrome coronavirus 2.

Within the evaluable immunogenicity population of the expanded cohort without evidence of infection up to 1 month after vaccination, GMTs against BA.1 were substantially elevated at 1 month compared with prestudy vaccination levels (Figure 3*A*). The highest GMT (95% CI) was observed with monovalent BA.1 60 µg (2828.3 [2259.8–3539.9]), whereas bivalent BA.1 60 µg and bivalent BA.1 30 μg were similar (1424.7 [1139.9–1780.8] and 1245.3 [1013.3–1530.3], respectively). Geometric mean fold rises (95% CI) from before to 1 month after study vaccination were higher with monovalent BA.1 60 µg (24.6 [19.3–31.4]) versus bivalent BA.1 60 µg (17.1 [13.7–21.4]) and bivalent BA.1 30 µg (15.4 [12.4–19.2]). Among those with or without evidence of prior infection, GMTs were highest with monovalent BA.1 60 µg followed by bivalent BA.1 60 µg and bivalent BA.1 30 µg (Figure 3*B*). Geometric mean fold rises were higher in those with negative (range, 16.8–27.0) versus positive (range, 4.7–8.4) baseline SARS-CoV-2 status.

In participants without evidence of infection up to 1 month after study vaccination, the percentages of participants achieving seroresponses in SARS-CoV-2 50% neutralizing titers 1 month after vaccination for Omicron BA.1 were 88.5%, 87.6%, and 95.6% with bivalent BA.1 60 µg, bivalent BA.1 30 µg, and monovalent BA.1 60 µg, respectively (Table 3). Results were similar to those with or without evidence of infection up to 1 month after study vaccination (81.8%, 82.2%, and 89.6%, respectively; Supplementary Table 2). Table 3 shows results of supportive analyses (GMRs for Omicron BA.1 neutralizing titers and differences in percentages of participants with seroresponses) among individuals without evidence of infection. Seroresponses were higher among those negative for SARS-CoV-2 at baseline for both the Omicron BA.1 (bivalent BA.1 60 µg, 89.5%; bivalent BA.1 30 µg, 89.1%; monovalent BA.1 60 µg, 96.1%) than those positive at baseline (55.9%, 57.6%, and 73.2%) (Supplementary Table 3).

**Table 3.** GMRs and Seroresponse Rates Among Participants Without Evidence of Infection for Omicron BA.1 Variant and Ancestral Strain in the Expanded Cohort (Evaluable Immunogenicity Population)

GMR and Seroresponse Rate	Participants 18-55 Years Old			Participants >55 Years Old [19]
	Bivalent BA.1 60 µg (n = 104)	Bivalent BA.1 30 µg (n = 105)	Monovalent BA.1 60 µg (n = 91)	Bivalent BA.1 30 µg (n = 178)
GMR (95% CI)^a,b,c^				
Omicron BA.1	1.68 (1.26–2.25)	1.47 (1.11–1.94)	3.34 (2.50–4.46)	1.56 (1.17–2.08)
Ancestral strain	1.21 (0.95–1.54)^d^	1.07 (0.85–1.35)	1.54 (1.20–1.98)	0.99 (0.82–1.20)
Seroresponse rate, % (95% CI)^e^				
Omicron BA.1	88.5 (80.7–93.9)	87.6 (79.8–93.2)	95.6 (89.1–98.8)	71.6 (64.2–78.3)
Ancestral strain	69.9 (60.1–78.5)^c^	64.8 (54.8–73.8)	70.3 (59.8–79.5)	50.0 (42.6–57.4)
Differences in seroresponse rates, % (95% CI)^a,f^				
Omicron BA.1	21.5 (10.7–32.0)	20.7 (9.8–31.3)	28.6 (18.9–38.4)	14.6 (4.0–24.9)
Ancestral strain	21.7 (8.6–34.0)^c^	16.5 (3.3–29.1)	22.1 (8.6–34.7)	NA

Data are shown for participants who had no serologic or virologic evidence (before the 1-month poststudy vaccination blood sample collection) of SARS-CoV-2 infection (ie, a negative N-binding antibody [serum] result at the study vaccination and the 1-m poststudy vaccination visits, a negative NAAT [nasal swab] result at the study vaccination visit and at any unscheduled visit before the 1-month poststudy vaccination blood sample collection), and had no medical history of COVID-19.

Abbreviations: CI, confidence inerval; COVID-19, coronavirus disease 2019; GMR, geometric mean ratio; NA, not available; NAAT, nucleic acid amplification test; SARS-CoV-2, severe acute respiratory syndrome coronavirus 2.

^a^Compared with a control group of participants aged >55 years from the same study who received bivalent BA.1 30 ug.

^b^GMRs and 2-sided 95% CIs were calculated by exponentiating the mean difference of the logarithms of the titers (vaccine group in the corresponding >55 years of age from the same study who received bivalent BA.1 30 ug) and the corresponding CI (based on the Student *t* distribution).

^c^GMRs for participants aged >55 years compared with GMRs achieved by participants who received BNT162b2 30 ug.

^d^The number of patients evaluable for ancestral strain was n = 103.

^e^Exact 2-sided CI based on the Clopper and Pearson method.

^f^The 2-sided CI based on the Miettinen and Nurminen method for the difference in proportions, expressed as a percentage.

#### Ancestral Strain

**Figure 4.**
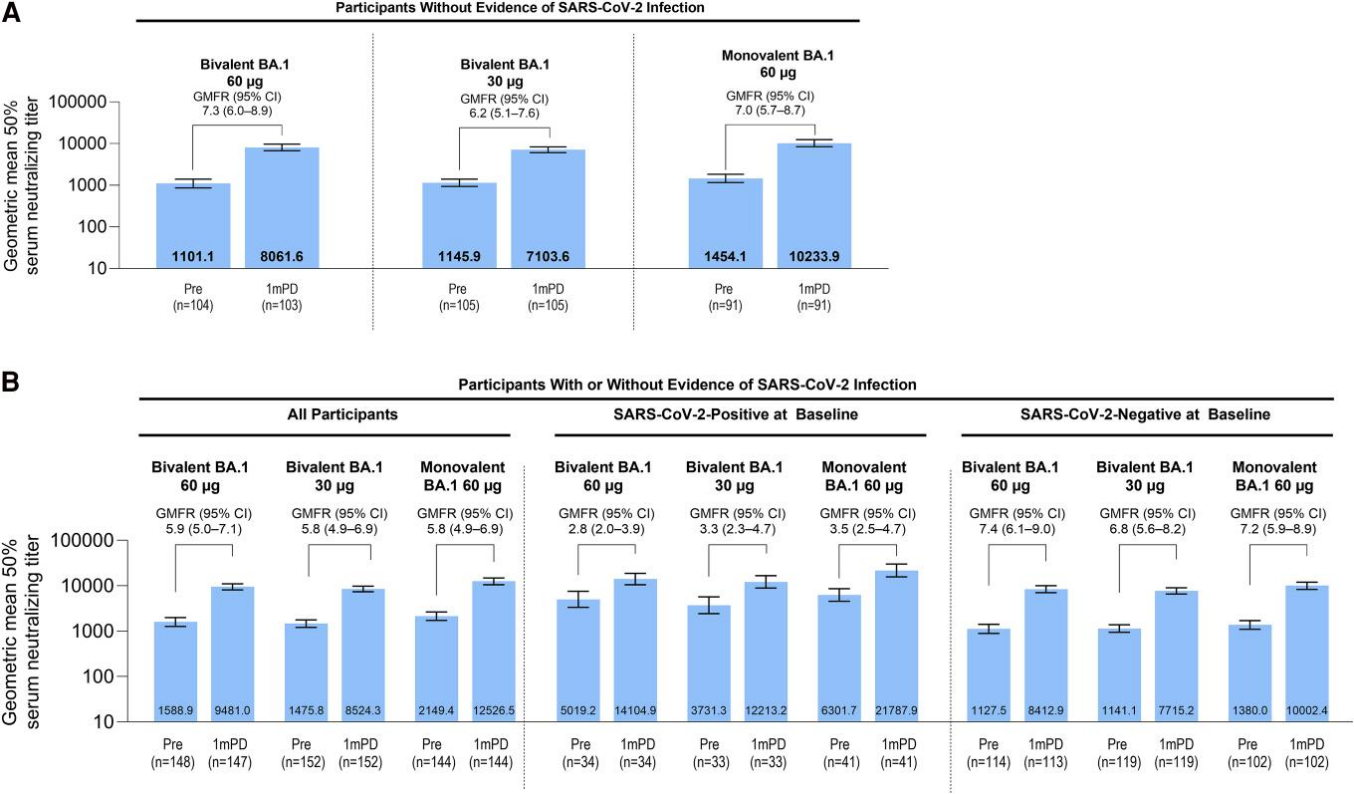
SARS-CoV-2 50% neutralization GMTs (95% CI) and GMFRs (95% CI) before and at 1 m after vaccination for the ancestral strain in expanded cohort participants without evidence of SARS-CoV-2 infection (*A*), those with or without evidence of SARS-CoV-2 infection and by baseline SARS-CoV-2 status (*B*) (evaluable immunogenicity population). For GMTs and GMFRs, assay results below the LLOQ were set to 0.5 × LLOQ in the analysis. Abbreviations: 1mPD, 1 m postdose; CI, confidence interval; GMFR, geometric mean fold rise; GMT, geometric mean titer; LLOQ, lower limit of quantitation; pre, before vaccination; SARS-CoV-2, severe acute respiratory syndrome coronavirus 2.

Across all vaccine groups in the expanded cohort evaluable immunogenicity population without evidence of infection, GMTs against the ancestral strain were substantially elevated at 1 month after versus before study vaccination (Figure 4*A*). Geometric mean fold rises (95% CI) were similar across all vaccine groups without evidence of infection up to 1 month after study vaccination: 7.3 (6.0–8.9) with bivalent BA.1 60 μg, 6.2 (5.1–7.6) with bivalent BA.1 30 μg, and 7.0 (5.7–8.7) with monovalent BA.1 60 μg. Regardless of baseline SARS-CoV-2 status, GMTs were highest with monovalent BA.1 60 µg followed by bivalent BA.1 60 µg and bivalent BA.1 30 µg (Figure 4*B*). Geometric mean fold rises were higher in those with a negative (range, 6.8–7.4) versus positive (range, 2.8–3.5) baseline SARS-CoV-2 status.

In participants without evidence of infection, the percentages of participants who achieved seroresponses 1 month after vaccination were 69.9%, 64.8%, and 70.3% with bivalent BA.1 60 µg, bivalent BA.1 30 µg, and monovalent BA.1 60 µg, respectively (Table 3). Results were similar in the overall population (ie, those with or without evidence of infection) up to 1 month after study vaccination (61.2%, 59.9%, and 63.9%, respectively) (Supplementary Table 2). Seroresponses were higher among those who were negative (bivalent BA.1 60 µg, 70.8%; bivalent BA.1 30 µg, 68.1%; monovalent BA.1 60 µg, 71.6%) versus positive (29.4%, 30.3%, and 46.3%) for SARS-CoV-2 at baseline (Supplementary Table 3).

#### Omicron BA.4/BA.5

**Figure 5.**
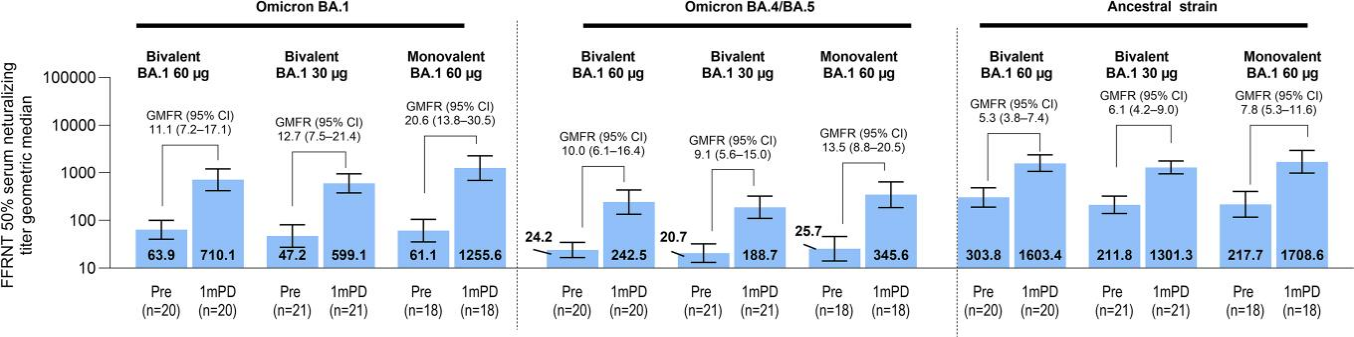
SARS-CoV-2 50% FFRNT GMTs (95% CI) and GMFRs (95% CI) for Omicron BA.1, Omicron BA.4/BA.5, and the ancestral strain in sentinel cohort participants without evidence of SARS-CoV-2 infection from the sentinel cohort (evaluable immunogenicity population). For GMTs and GMFRs, assay results below the LLOQ were set to 0.5 × LLOQ in the analysis. Abbreviations: 1mPD, 1 m postdose; CI, confidence inerval; FFRNT, fluorescent focus reduction neutralization test; GMFR, geometric mean fold rise; GMT, geometric mean titer; LLOQ, lower limit of quantitation; pre, before vaccination; SARS-CoV-2, severe acute respiratory syndrome coronavirus 2.

Additional sublineages were investigated only in the sentinel cohort. In the evaluable immunogenicity population in participants without prior evidence of infection up to 1 month after study vaccination, GMTs for the Omicron BA.4/BA.5 subvariant at 1 month after versus before vaccination were numerically higher in all groups, although lower than respective responses to BA.1 and the ancestral strain (Figure 5).

### COVID-19 Surveillance

In the expanded cohort, 185 participants (19.2%) reported COVID-19 cases through a median of 6.3 (range, 1.6–7.8) months of follow-up, including 95 cases (19.7%) with bivalent BA.1 60 µg, 32 (20.1%) with bivalent BA.1 30 µg, and 58 (18.1%) with monovalent BA.1 60 µg. No severe cases of COVID-19 occurred after study vaccination. Of 185 identified cases, 14 (7.6%) had insufficient quantity or were not sequenced and therefore of unknown lineage, and most were B.1.1.529 (Omicron) sublineages, including 17 (9.2%) BA.5.2.1 cases and 13 (7.0%) cases each of BA.5 and BA5.2 sublineages.

## DISCUSSION

In this analysis of adults 18‒55 years old who previously received three 30 μg doses of BNT162b2, monovalent and bivalent Omicron BA.1–adapted BNT162b2 vaccines induced neutralization titers against Omicron BA.1, and robust neutralization titers were maintained against ancestral strain. The monovalent and bivalent vaccines also induced neutralization titers against Omicron BA.4/BA.5, although reduced compared with BA.1. Immune responses against BA.1 were higher with monovalent BA.1 60 µg relative to bivalent BA.1 60 and 30 µg, which showed similar responses. Robust immune responses were observed for all vaccines regardless of previous SARS-CoV-2 infection.

The observed safety profiles of Omicron BA.1–adapted vaccines administered as a fourth dose were consistent with the known safety profile of the original 30 μg dose of BNT162b2 [3, 18, 25]. The slightly higher rates of AEs observed with the 60 µg dose of monovalent BA.1–adapted vaccine versus the 30 µg dose are consistent with findings in older participants (≥55 years) from this trial [19].

Since November 2021, Omicron sublineages have dominated the SARS-CoV-2 epidemiologic landscape [26]. The bivalent Omicron BA.1–adapted vaccine used here was authorized by the European Medicines Agency (EMA) as an additional dose in persons ≥12 years old. BA.1‒adapted vaccines offered greater protection against BA.1 strains compared with BA.2 and BA.4/BA.5 [19]. Subsequently, new Omicron sublineages descended from BA.2 and BA.4/BA.5 have emerged [23], including JN.1, a descendant of BA.2.86, predominant globally since April 2024 [27, 28]. In response to the Omicron BA.4 and BA.5 sublineages, the US Food and Drug Administration and EMA authorized additional bivalent Omicron BA.4/BA.5–adapted BNT162b2 doses in persons ≥5 years old in late 2022 [29–31]. In participants >55 years old, a booster dose of BA.4/BA.5–adapted vaccine produced neutralizing titers against BA.5–derived (BA.4.6, BQ.1.1, XBB.1.5, and XBB.1.16) and BA.2–derived (BA.2.75.2) sublineages that were higher than a fourth original BNT162b2 dose regardless of SARS-CoV-2 infection history [23]. Real-world studies conducted in 2022 and 2023 reported that bivalent BA.4/5 or BA.1 vaccines reduced rates of COVID-19–related hospitalizations [32, 33]. In response to the continued evolution of SARS-CoV-2, before the 2023/2024 winter season, a monovalent XBB.1.5–adapted vaccine was approved in the United States and other countries [31, 34], with similarly improved protection against circulating strains [35, 36]. Vaccines will likely need to be updated periodically to match circulating strains.

Limitations of this study are lack of longer-term follow-up to assess the duration of immune response and safety, US-based and predominantly White non-Hispanic/Latino trial population, and exclusion of immunocompromised persons. Also, because the Omicron BA.1–adapted vaccines were not compared with the original BNT162b2 vaccine in the 18‒55-year-old cohort, we cannot speculate on the presence or absence of immune imprinting, a phenomenon recently reported in studies of additional doses of Omicron BA.4/BA.5 and BA.5 bivalent vaccines [37, 38].

In conclusion, the tolerability of monovalent (30 µg dose) and bivalent (30 and 60 µg doses) Omicron BA.1‒adapted vaccines was favorable, and safety profiles were consistent with that of original BNT162b2. Robust immune responses against Omicron BA.1 and ancestral strain were observed with both monovalent and bivalent Omicron BA.1–modified vaccines administered as a fourth dose to BNT162b2–experienced participants 18‒55 years old. These results highlight that variant-adapted vaccines are safe, induce robust immune responses, and may protect against closely matched strains.

## Supplementary Material

ciag026_Supplementary_Data

## Supplementary Data

Supplementary materials are available at *Clinical Infectious Diseases* online. Consisting of data provided by the authors to benefit the reader, the posted materials are not copyedited and are the sole responsibility of the authors, so questions or comments should be addressed to the corresponding author.

## References

[1] World Health Organization . COVID-19 advice for the public: getting vaccinated. Available at: https://www.who.int/emergencies/diseases/novel-coronavirus-2019/covid-19-vaccines/advice#:∼:text=Currently%20approved%20COVID%2D19%20vaccines,more%20beneficial%20than%20delaying%20vaccination. Accessed 18 March 2024.

[2] COMIRNATY® (COVID-19 Vaccine, mRNA). Full Prescribing Information, BioNTech Manufacturing GmbH and Pfizer Inc, New York, NY 2025. Available at: https://labeling.pfizer.com/ShowLabeling.aspx?id=16351&format=pdf&cmp=171631588317&utm_source=MICROSOFT&utm_medium=paidsearch&pid=Brand&utm_term=pfizer%20covid-19%20vaccine%20primary%20series&network=o&gclid=2fe274c37f0c1b7eafe11db9ffc2aeab&gclsrc=3p.ds&msclkid=2fe274c37f0c1b7eafe11db9ffc2aeab. Accessed 1 February 2026.

[3] Polack FP, Thomas SJ, Kitchin N, et al Safety and efficacy of the BNT162b2 mRNA COVID-19 vaccine. N Engl J Med 2020; 383:2603–15. 33301246 10.1056/NEJMoa2034577 PMC7745181

[4] Frenck RW Jr, Klein NP, Kitchin N, et al Safety, immunogenicity, and efficacy of the BNT162b2 Covid-19 vaccine in adolescents. N Engl J Med 2021; 385:239–50. 34043894 10.1056/NEJMoa2107456 PMC8174030

[5] Gruell H, Vanshylla K, Tober-Lau P, et al mRNA booster immunization elicits potent neutralizing serum activity against the SARS-CoV-2 Omicron variant. Nat Med 2022; 28:477–80. 35046572 10.1038/s41591-021-01676-0 PMC8767537

[6] Haas EJ, Angulo FJ, McLaughlin JM, et al Impact and effectiveness of mRNA BNT162b2 vaccine against SARS-CoV-2 infections and COVID-19 cases, hospitalisations, and deaths following a nationwide vaccination campaign in Israel: an observational study using national surveillance data. Lancet 2021; 397:1819–29. 33964222 10.1016/S0140-6736(21)00947-8 PMC8099315

[7] Lopez Bernal J, Andrews N, Gower C, et al Effectiveness of Covid-19 vaccines against the B.1.617.2 (Delta) variant. N Engl J Med 2021; 385:585–94. 34289274 10.1056/NEJMoa2108891 PMC8314739

[8] Singer SR, Angulo FJ, Swerdlow DL, et al Effectiveness of BNT162b2 mRNA COVID-19 vaccine against SARS-CoV-2 variant Beta (B.1.351) among persons identified through contact tracing in Israel: a prospective cohort study. EClinicalMedicine 2021; 42:101190. 34870134 10.1016/j.eclinm.2021.101190 PMC8628463

[9] Zheng C, Shao W, Chen X, Zhang B, Wang G, Zhang W. Real-world effectiveness of COVID-19 vaccines: a literature review and meta-analysis. Int J Infect Dis 2022; 114:252–60. 34800687 10.1016/j.ijid.2021.11.009 PMC8595975

[10] Shao W, Chen X, Zheng C, et al Effectiveness of COVID-19 vaccines against SARS-CoV-2 variants of concern in real-world: a literature review and meta-analysis. Emerg Microbes Infect 2022; 11:2383–92. 36069511 10.1080/22221751.2022.2122582 PMC9542696

[11] Andrews N, Stowe J, Kirsebom F, et al COVID-19 vaccine effectiveness against the omicron (B.1.1.529) variant. N Engl J Med 2022; 386:1532–46. 35249272 10.1056/NEJMoa2119451 PMC8908811

[12] Gazit S, Saciuk Y, Perez G, Peretz A, Pitzer VE, Patalon T. Short term, relative effectiveness of four doses versus three doses of BNT162b2 vaccine in people aged 60 years and older in Israel: retrospective, test negative, case-control study. BMJ 2022; 377:e071113. 35609888 10.1136/bmj-2022-071113 PMC9127435

[13] Tartof SY, Slezak JM, Puzniak L, et al Durability of BNT162b2 vaccine against hospital and emergency department admissions due to the Omicron and Delta variants in a large health system in the USA: a test-negative case-control study. Lancet Respir Med 2022; 10:689–99. 35468336 10.1016/S2213-2600(22)00101-1 PMC9033225

[14] Li Q, Zhang M, Liang Z, et al Antigenicity comparison of SARS-CoV-2 omicron sublineages with other variants contained multiple mutations in RBD. MedComm (2020) 2022; 3:e130. 35434713 10.1002/mco2.130 PMC8994617

[15] US Food and Drug Administration . COVID-19 vaccines (2025-2026 Formula) for use in the United States beginning in fall 2025. Available at: https://www.fda.gov/vaccines-blood-biologics/industry-biologics/covid-19-vaccines-2025-2026-formula-use-united-states-beginning-fall-2025. Accessed 2 February 2026.

[16] European Medicines Agency . Procedural guidance for variant strain(s) update to vaccines intended for protection against human coronavirus. Available at: https://www.ema.europa.eu/en/documents/regulatory-procedural-guideline/procedural-guidance-variant-strains-update-vaccines-intended-protection-against-human-coronavirus_en.pdf. Accessed 22 September 2022.

[17] Tulimilli SV, Dallavalasa S, Basavaraju CG, et al Variants of severe acute respiratory syndrome coronavirus 2 (SARS-CoV-2) and vaccine effectiveness. Vaccines (Basel) 2022; 10:1751. 36298616 10.3390/vaccines10101751 PMC9607623

[18] Moreira ED Jr, Kitchin N, Xu X, et al Safety and efficacy of a third dose of BNT162b2 COVID-19 vaccine. N Engl J Med 2022; 386:1910–21. 35320659 10.1056/NEJMoa2200674 PMC9006787

[19] Winokur P, Gayed J, Fitz-Patrick D, et al Bivalent Omicron BA.1-adapted BNT162b2 booster in adults older than 55 years. N Engl J Med 2023; 388:214–27. 36652353 10.1056/NEJMoa2213082 PMC9933930

[20] Tuekprakhon A, Nutalai R, Dijokaite-Guraliuc A, et al Antibody escape of SARS-CoV-2 Omicron BA.4 and BA.5 from vaccine and BA.1 serum. Cell 2022; 185:2422–33.e13. 35772405 10.1016/j.cell.2022.06.005 PMC9181312

[21] Kurhade C, Zou J, Xia H, et al Neutralization of omicron BA.1, BA.2, and BA.3 SARS-CoV-2 by 3 doses of BNT162b2 vaccine. Nat Commun 2022; 13:3602. 35739094 10.1038/s41467-022-30681-1 PMC9225806

[22] Kurhade C, Zou J, Xia H, et al Neutralization of Omicron sublineages and Deltacron SARS-CoV-2 by three doses of BNT162b2 vaccine or BA.1 infection. Emerg Microbes Infect 2022; 11:1828–32. 35792746 10.1080/22221751.2022.2099305 PMC9331225

[23] Zou J, Kurhade C, Patel S, et al Neutralization of BA.4-BA.5, BA.4.6, BA.2.75.2, BQ.1.1, and XBB.1 with bivalent vaccine. N Engl J Med 2023; 388:854–7. 36734885 10.1056/NEJMc2214916 PMC9891359

[24] Zou J, Xia H, Xie X, et al Neutralization against Omicron SARS-CoV-2 from previous non-omicron infection. Nat Commun 2022; 13:852. 35140233 10.1038/s41467-022-28544-w PMC8828871

[25] Thomas SJ, Moreira ED Jr, Kitchin N, et al Safety and efficacy of the BNT162b2 mRNA Covid-19 vaccine through 6 months. N Engl J Med 2021; 385:1761–73. 34525277 10.1056/NEJMoa2110345 PMC8461570

[26] Velavan TP, Ntoumi F, Kremsner PG, Lee SS, Meyer CG. Emergence and geographic dominance of Omicron subvariants XBB/XBB.1.5 and BF.7 - the public health challenges. Int J Infect Dis 2023; 128:307–9. 36681145 10.1016/j.ijid.2023.01.024 PMC9850647

[27] GISAID . Tracking of hCoV-19 variants. Available at: https://gisaid.org/hcov19-variants/. Accessed 25 March 2024.

[28] Centers for Disease Control . COVID-19 activity increases as prevalence of JN.1 variant continues to rise. Available at: https://www.cdc.gov/ncird/whats-new/JN.1-update-2024-01-05.html#print. Accessed 9 April 2024.

[29] US Food and Drug Administration . Coronavirus (COVID-19) Update: FDA Authorizes Moderna, Pfizer-BioNTech Bivalent COVID-19 vaccines for use as a booster dose. Available at: https://web.archive.org/web/20221028135528/https://www.fda.gov/news-events/press-announcements/coronavirus-covid-19-update-fda-authorizes-moderna-pfizer-biontech-bivalent-covid-19-vaccines-use. Accessed 2 February 2026.

[30] US Food and Drug Administration . Coronavirus (COVID-19) Update: FDA authorizes first oral antiviral for treatment of COVID-19. Available at: https://web.archive.org/web/20221130214837/https://www.fda.gov/news-events/press-announcements/coronavirus-covid-19-update-fda-authorizes-moderna-and-pfizer-biontech-bivalent-covid-19-vaccines. Accessed 2 February 2026.

[31] European Medicines Agency . Adapted vaccine targeting BA.4 and BA.5Omicron variants and original SARS-CoV-2recommended for approval. Available at: https://www.ema.europa.eu/en/news/adapted-vaccine-targeting-ba4-ba5-omicron-variants-original-sars-cov-2-recommended-approval. Accessed 14 November 2023.

[32] Andersson NW, Thiesson EM, Baum U, et al Comparative effectiveness of bivalent BA.4-5 and BA.1 mRNA booster vaccines among adults aged ≥50 years in Nordic countries: nationwide cohort study. BMJ 2023; 382:e075286. 37491022 10.1136/bmj-2022-075286 PMC10364194

[33] Kirsebom FCM, Andrews N, Stowe J, Ramsay M, Lopez Bernal J. Duration of protection of ancestral-strain monovalent vaccines and effectiveness of bivalent BA.1 boosters against COVID-19 hospitalisation in England: a test-negative case-control study. Lancet Infect Dis 2023; 23:1235–43. 37453440 10.1016/S1473-3099(23)00365-1

[34] Food and Drug Administration . Recommendation for the 2023-2024 Formula of COVID-19 vaccines in the U.S. Available at: https://www.fda.gov/media/169591/download. Accessed 2 February 2026.

[35] Hansen CH, Moustsen-Helms IR, Rasmussen M, Søborg B, Ullum H, Valentiner-Branth P. Short-term effectiveness of the XBB.1.5 updated COVID-19 vaccine against hospitalisation in Denmark: a national cohort study. Lancet Infect Dis 2024; 24:e73–4. 38190834 10.1016/S1473-3099(23)00746-6

[36] Link-Gelles R, Ciesla AA, Mak J, et al Early estimates of updated 2023-2024 (monovalent XBB.1.5) COVID-19 vaccine effectiveness against symptomatic SARS-CoV-2 infection attributable to co-circulating omicron variants among immunocompetent adults - increasing community access to testing program, United States, September 2023-January 2024. MMWR Morb Mortal Wkly Rep 2024; 73:77–83. 38300853 10.15585/mmwr.mm7304a2 PMC10843065

[37] Collier AY, Miller J, Hachmann NP, et al Immunogenicity of BA.5 bivalent mRNA vaccine boosters. N Engl J Med 2023; 388:565–7. 36630611 10.1056/NEJMc2213948 PMC9847505

[38] Wang Q, Bowen A, Valdez R, et al Antibody response to Omicron BA.4-BA.5 bivalent booster. N Engl J Med 2023; 388:567–9. 36630643 10.1056/NEJMc2213907 PMC9847504

