# Supplementary material for: Safety and Immunogenicity of a Fourth Dose of Omicron BA.1–Adapted BNT162b2 COVID-19 Vaccines in Adults 18–55 Years Old
Source: Clin Infect Dis. 2026 Jan 21;82(5):e1083–92. doi: 10.1093/cid/ciag026 (PMC13189652; doi:10.1093/cid/ciag026)
Supplement: ciag026_Supplementary_Data [file ciag026_supplementary_data.docx]

# Supplementary Material

**Immunogenicity and Safety of Omicron-BA.1-Adapted BNT162b2 COVID-19 Boosters in Adults 18‒55 Years Old**

**Authors:** Patricia Winokur, MD,^1^ Oyeniyi Diya, MD,^2^ David Fitz-Patrick, MD,^3^ Michael Dever, MD,^4^ Juleen Gayed, MBBS, MSc, FFPM,^2^* Stephen Lockhart, DM,^2^ Xia Xu, PhD,^5^ Ying Zhang, PhD,^6^ Vishva Bangad, MS,^5^ L Tyler Wadsworth, MD, FACSM, FAMSSM,^7^ Kevin Cannon, MD,^8^ Jose F Cardona, MD, JD, MBA-HA,^9^ Lisa Usdan, MD,^10^ John Ginis, BS,^5^ Federico J Mensa, MD,^11^ Jing Zou, PhD,^12^ Xuping Xie, PhD,^12^ Claire Lu,^6^ PhD, Sandra Buitrago, MS,^6^ Ingrid L Scully, PhD,^6^ David Cooper, PhD,^6^ Kenneth Koury, PhD,^6^ Kathrin U Jansen, PhD,^6^ Ӧzlem Türeci, MD,^11^ Uğur Şahin, MD,^11^ Kena A Swanson, PhD,^6^ William C Gruber, MD,^6^ Nicholas Kitchin, MD,^2^ on behalf of the C4591031 Clinical Trial Group.

**Affiliations:** ^1^Division of Infectious Diseases, Carver College of Medicine, University of Iowa, Iowa City, IA, USA; ^2^Vaccine Research and Development, Pfizer Ltd, Hurley, UK; ^3^East-West Medical Research Institute, Honolulu, HI, USA; ^4^Clinical Neuroscience Solutions, Orlando, FL, USA; ^5^Vaccine Research and Development, Pfizer Inc, Collegeville, PA, USA; ^6^Vaccine Research and Development, Pfizer, Pearl River, NY, USA; ^7^Sundance Clinical Research, St Louis, MO, USA; ^8^PMG Research of Wilmington, Wilmington, NC, USA; ^9^Indago Research & Health Center, Inc, Hialeah, FL, USA; ^10^CNS Healthcare, Memphis, TN, USA; ^11^BioNTech, Mainz, Germany; ^12^University of Texas Medical Branch, Galveston, TX, USA

***Corresponding author:** Juleen Gayed, MBBS, MSc, FFPM

Pfizer Vaccine Clinical Research & Development

Horizon Building, Honey Lane, Hurley, SL6 6RJ, UK

Email: juleen.gayed@pfizer.com

Telephone: (+44) 1628 515816

## Supplementary Methods

### Sentinel Cohort

The sentinel cohort comprised 90 participants, with 30 participants each randomized to a 30-µg or 60-µg dose of bivalent BA.1-adapted BNT162b2 or a 60-µg dose of monovalent BA.1-adapted BNT162b2. An independent review committee confirmed that all safety data (ie, adverse events, reactogenicity e-diary data, and troponin levels) through Day 7 from the sentinel cohort were acceptable before enrolling the expanded cohort.

### Vaccine Formulation and Administration

Bivalent BA.1 60 µg or 30 µg was provided as preformulated suspensions for injection in a single glass vial with no dilution required, and monovalent BA.1 60 µg was provided in a glass vial requiring dilution. Participants in each group received a single dose intramuscularly (deltoid, nondominant arm) on study Day 1.

### Definition of Without Evidence of SARS-CoV-2 Infection

Participants who had no serological or virological evidence (before the 1-month poststudy vaccination blood sample collection) of SARS-CoV-2 infection were defined as having negative N-binding antibody (serum) result at the study vaccination and the 1-month poststudy vaccination visits, a negative nucleic acid amplification test (NAAT; via nasal swab) at the study vaccination visit and at any unscheduled visit before the 1-month poststudy vaccination blood sample collection, and had no medical history of COVID-19.

### Definitions of COVID-19 cases

If, at any time during the study, a participant developed acute respiratory illness he or she was considered to potentially have COVID-19 illness. Assessment of COVID-19 included nasal swab, which was tested at a central laboratory using reverse transcription–polymerase chain reaction (RT-PCR), or other equivalent NAAT to detect SARS-CoV-2. In addition, clinical information and results from local standard-of-care tests were assessed. The central laboratory NAAT result was used for the case definition, unless no result was available from the central laboratory, in which case a local NAAT result could be used.

Confirmed COVID-19 was defined as presence of at least 1 of the following symptoms and SARS-CoV-2 NAAT-positive during, or within 4 days before or after, the symptomatic period, either at the central laboratory or at a local testing facility (using an acceptable test): fever, new or increased cough, new or increased shortness of breath, chills, new or increased muscle pain, new loss of taste or smell, sore throat, diarrhea; vomiting. Confirmed severe COVID-19 was assessed based on either the Food and Drug Administration (FDA) [1] and Centers for Disease Control and Prevention (CDC) [2] definitions. For the FDA criteria, severe COVID-19 was based on confirmed infection and at least one of the following: clinical signs at rest indicative of severe systemic illness (respiration rate ≥30 breaths per minute, heart rate ≥125 beats per minute, SpO2 ≤93% on room air at sea level, or partial pressure of oxygen [arterial]/fraction of inspired oxygen <300 mm Hg);respiratory failure (defined as needing high-flow oxygen, noninvasive ventilation, mechanical ventilation, or extracorporeal membrane oxygenation); evidence of shock (systolic blood pressure <90 mm Hg, diastolic blood pressure <60 mm Hg, or requiring vasopressors); significant acute renal, hepatic, or neurologic dysfunction; admission to an intensive care unit (ICU); or death [1]. For the CDC criteria, severe COVID-19 was based on confirmed infection and at least one of the following: hospitalization, admission to the ICU, intubation or mechanical ventilation, or death [2].

## Supplementary Results

### Sentinel Cohort Demographics

In the sentinel cohort, all 90 randomized participants (30/treatment arm) were vaccinated. The cutoff date was July 11, 2022 for the 1-month data and was March 24, 2023 for the 6-month data. All but 5 completed the study. There were no withdrawals from the safety population; 82/90 participants (91.1%) had their 1-month postvaccination blood draw 28‒35 days after study vaccination. The median time from Dose 3 of BNT162b2 to study vaccination was 8.2 months.

Patient demographics and baseline clinical characteristics (**Supplementary Table S4**) were similar between treatment groups. Patterns of local reactions and systemic events in the sentinel cohort were generally similar to those in the expanded cohort (**Supplementary Figure S1**). Adverse event rates across all study vaccines in the sentinel cohort were slightly higher compared with those in the expanded cohort, except for severe and life-threatening adverse events (**Supplementary Table S5**).

The evaluable immunogenicity population comprised 85 participants in the sentinel cohort (bivalent BA.1 60 µg, n=30; bivalent BA.1 30 µg, n=29; monovalent BA.1 60 µg, n=26); there were 59 participants without evidence of infection up to 1 month after vaccination (bivalent BA.1 60 µg, n=20; bivalent BA.1 30 µg, n=21; monovalent BA.1 60 µg, n=18). In the evaluable immunogenicity population without prior evidence of infection up to 1 month after study vaccination, Omicron BA.1 Geometric mean titers (GMTs) at 1 month after versus before study vaccination were substantially elevated across all vaccine groups, with the monovalent BA.1 60-µg group showing the best responses (**Figure 5**). Geometric mean fold rises (GMFRs) were high for all vaccine groups: 11.1 in the bivalent BA.1 60-µg group, 12.7 in the bivalent BA.1 30-µg group, and 20.6 in the monovalent BA.1 60-µg group. Similar GMT and GMFR results were observed in those with or without prior evidence of infection up to 1 month after study vaccination (**Supplementary Figure S2**).

The GMTs for the ancestral strain in the sentinel cohort without prior evidence of infection up to 1 month after study vaccination were also substantially higher at 1 month after versus before vaccination across all vaccine groups (**Figure 5**). GMFRs were high for all groups: 5.3, 6.1, and 7.8 in the bivalent BA.1 60-µg, bivalent BA.1 30-µg, and monovalent BA.1 60-µg groups, respectively. Similar GMT and GMFR results were observed in those with or without prior evidence of infection up to 1 month after study vaccination (**Supplementary Figure S2**).

**Figure S1.** Local reactions (A) and systemic events (B) reported within 7 days of vaccination in the sentinel cohort (safety population). Data are presented for the safety population (all participants who received the study intervention) in the expanded cohort. Severity grading of the specific local reactions and systemic events is provided in Table S1. Bars represent the 95% CIs, and numbers above the bars indicate the percentage of participants in each group reporting any severity for the specified event. The bivalent BA.1 60**-**μg group included participants who received 30 μg BNT162b2-Omi.BA.1 + 30 μg BNT162b (n=30), the bivalent BA.1 30**-**μg group included those who received 15 μg BNT162b2-Omi.BA.1 + 15 μg BNT162b2 (n=30), and the monovalent BA.1 60**-**μg included those who received 60 μg BNT162b2-Omi.BA.1 (n=30).

**Figure S2.** SARS-CoV-2 50 fluorescent focus reduction neutralization test GMTs (95% CI) and GMFRs (95% CI) for Omicron BA.1, Omicron BA.4/BA.5, and the ancestral strains in participants with or without evidence of SARS-CoV-2 infection from the sentinel cohort**.** Data are shown for participants regardless of prior evidence of infection up to 1 month after study vaccination. GMTs and 2-sided 95% CIs were calculated by exponentiating the mean logarithm of the titers and the corresponding CIs (based on the Student *t* distribution); assay results below the LLOQ were set to 0.5 × LLOQ. GMFRs and 2-sided 95% CIs were calculated by exponentiating the mean logarithm of fold rises and the corresponding CIs (based on the Student *t* distribution); assay results below the LLOQ were set to 0.5 × LLOQ in the analysis. Abbreviations: 1mPD, 1-month postdose; FFRNT, fluorescent focus reduction neutralization test; GMFR, geometric mean fold rise; GMT, geometric mean titer; LLOQ, lower limit of quantitation.

## Table S1. Severity Grading of Local Reactions and Systemic Events

| **Local Reaction or Systemic Event** | **Mild (Grade 1)** | **Moderate (Grade 2)** | **Severe (Grade 3)** | **Potentially Life-Threatening (Grade 4)** |
| --- | --- | --- | --- | --- |
| Local reaction |  |  |  |  |
| Pain at the injection site | Does not interfere with activity | Interferes with activity | Prevents daily activity | ED visit or hospitalization for severe pain |
| Redness | >2.0 to 5.0 cm (5‒10 measuring device units) | >5.0 to 10.0 cm (11‒20 measuring device units) | >10.0 cm (≥21 measuring device units) | Necrosis or exfoliative dermatitis |
| Swelling | >2.0 to 5.0 cm (5‒10 measuring device units) | >5.0 to 10.0 cm (11‒20 measuring device units) | >10.0 cm (≥21 measuring device units) | Necrosis |
| Systemic event |  |  |  |  |
| Vomiting | 1‒2 times in 24 hours | >2 times in 24 hours | Requires IV hydration | ED visit or hospitalization for hypotensive shock |
| Diarrhea | 2‒3 loose stools in 24 hours | 4‒5 loose stools in 24 hours | ≥6 loose stools in 24 hours | ED visit or hospitalization for severe diarrhea |
| Headache | Does not interfere with activity | Some interference with activity | Prevents daily routine activity | ED visit or hospitalization for severe headache |
| Fatigue/tiredness | Does not interfere with activity | Some interference with activity | Prevents daily routine activity | ED visit or hospitalization for severe fatigue |
| Chills | Does not interfere with activity | Some interference with activity | Prevents daily routine activity | ED visit or hospitalization for severe chills |
| New/worsened muscle pain | Does not interfere with activity | Some interference with activity | Prevents daily routine activity | ED visit or hospitalization for severe new/worsened muscle pain |
| New/worsened joint pain | Does not interfere with activity | Some interference with activity | Prevents daily routine activity | ED visit or hospitalization for severe new/worsened joint pain |

Abbreviations: ED, emergency department; IV, intravenous.

## Table S2. GMRs and Seroresponse Rates for Omicron BA.1 Variant and Ancestral Strain in the Expanded Cohort (Evaluable Immunogenicity Population)

| **GMR and Seroresponse Rate** | **Bivalent BA.1 60 µg (n=148)** | **Bivalent BA.1 30 µg (n=152)** | **Monovalent BA.1 60 µg (n=144)** |
| --- | --- | --- | --- |
| GMR (95% CI)^a,b^ |  |  |  |
| Omicron BA.1 | 1.68 (1.28–2.21) | 1.52 (1.17–1.98) | 3.63 (2.78–4.76) |
| Ancestral strain | 1.23 (0.99–1.53)^c^ | 1.11 (0.90–1.37) | 1.63 (1.29–2.05) |
| Seroresponse rates, % (95% CI)^d^ |  |  |  |
| Omicron BA.1 | 81.8 (74.6–87.6) | 82.2 (75.2–88.0) | 89.6 (83.4–94.1) |
| Ancestral strain | 61.2 (52.8–69.1)^c^ | 59.9 (51.6–67.7) | 63.9 (55.5–71.7) |
| Differences in seroresponse rates, % (95% CI)^e^ |  |  |  |
| Omicron BA.1 | 19.7 (9.4–29.8) | 20.2 (10.0–30.3) | 27.5 (17.9–37.0) |
| Ancestral strain | 15.6 (4.0–26.9)^c^ | 14.3 (2.7–25.5) | 18.3 (6.6–29.5) |

Abbreviations: GMR, geometric mean ratio.

Data are shown for participants regardless of prior evidence of infection up to 1 month after study vaccination.

^a^Compared with a control group of participants aged >55 years from the same study who received bivalent BA.1 30 ug.

^b^GMRs and 2-sided 95% CIs were calculated by exponentiating the mean difference of the logarithms of the titers (vaccine group in the corresponding >55 years of age from the same study who received bivalent BA.1 30 ug) and the corresponding CI (based on the Student *t* distribution).

^c^The number of patients evaluable for ancestral strain was n=147.

^d^Exact 2-sided CI based on the Clopper and Pearson method.

^e^The 2-Sided CI based on the Miettinen and Nurminen method for the difference in proportions, expressed as a percentage.

## Table S3. Seroresponse Rates by Baseline SARS-CoV-2 Status for Omicron BA.1 Variant and Ancestral Strain in the Expanded Cohort (Evaluable Immunogenicity Population)

| **Number of Participants and Seroresponse Rates** | **Bivalent BA.1 60 µg** | **Bivalent BA.1 30 µg** | **Monovalent BA.1 60 µg** |
| --- | --- | --- | --- |
| **Baseline SARS-CoV-2 Positive** | **n=34** | **n=33** | **n=41** |
| Seroresponse rates, % (95% CI)^a^ |  |  |  |
| Omicron BA.1 | 55.9 (37.9–72.8) | 57.6 (39.2–74.5) | 73.2 (57.1–85.8) |
| Ancestral strain | 29.4 (15.1–47.5) | 30.3 (15.6–48.7) | 46.3 (30.7–62.6) |
| **Baseline SARS-CoV-2 Negative** | **n=114** | **n=119** | **n=102** |
| Seroresponse rates, % (95% CI)^a^ |  |  |  |
| Omicron BA.1 | 89.5 (82.3–94.4) | 89.1 (82.0–94.1) | 96.1 (90.3–98.9) |
| Ancestral strain | 70.8 (61.5–79.0)^b^ | 68.1 (58.9–76.3) | 71.6 (61.8–80.1) |

Data are shown for participants regardless of prior evidence of infection up to 1 month after study vaccination.

^a^Exact 2-sided CI based on the Clopper and Pearson method.

^b^The number of patients evaluable for ancestral strain was n=113.

## Table S4. Patient Demographics and Baseline Clinical Characteristics (Sentinel Cohort Safety Population)

| **Characteristic** | **Sentinel Cohort (N=90)** | | |
| --- | --- | --- | --- |
|  | **Bivalent  BA.1 60 µg (n=30)** | **Bivalent  BA.1 30 µg (n=30)** | **Monovalent  BA.1 60 µg (n=30)** |
| Sex, n (%) |  |  |  |
| Male | 16 (53.3) | 13 (43.3) | 15 (50.0) |
| Female | 14 (46.7) | 17 (56.7) | 15 (50.0) |
| Age (range) at vaccination, median, years | 41.0 (18–55) | 41.5 (22–55) | 46.5 (18–55) |
| Race, n (%) |  |  |  |
| White | 25 (83.3) | 24 (80.0) | 26 (86.7) |
| Black/African American | 2 (6.7) | 3 (10.0) | 1 (3.3) |
| Asian | 2 (6.7) | 3 (10.0) | 3 (10.0) |
| Other^a^ | 1 (3.3) | 0 | 0 |
| Not reported | 0 | 0 | 0 |
| Ethnicity, n (%) |  |  |  |
| Hispanic/Latino | 3 (10.0) | 4 (13.3) | 3 (10.0) |
| Non-Hispanic/non-Latino | 27 (90.0) | 26 (86.7) | 27 (90.0) |
| Not reported | 0 | 0 | 0 |
| Baseline SARS-CoV-2 status, n (%) |  |  |  |
| Positive^b^ | 7 (23.3) | 7 (23.3) | 9 (30.0) |
| Positive NAAT | 0 | 1 (3.3) | 2 (6.7) |
| Negative^c^ | 23 (76.7) | 23 (76.7) | 21 (70.0) |
| Missing | 0 | 0 | 0 |
| Median time since prior receipt of BNT162b2 Dose 3 (range), months | 8.2 (5.5–11.5) | 7.9 (5.7–11.5) | 8.2 (4.3–11.5) |
| Time since prior receipt of BNT162b2 Dose 3, months, n (%) |  |  |  |
| <5 | 0 | 0 | 1 (3.3) |
| ≥5 to <7 | 5 (16.7) | 4 (13.3) | 4 (13.3) |
| ≥7 to <9 | 18 (60.0) | 18 (60.0) | 19 (63.3) |
| ≥9 to <11 | 0 | 1 (3.3) | 3 (10.0) |
| ≥11 to <12 | 7 (23.3) | 7 (23.3) | 3 (10.0) |
| >12 | 0 | 0 | 0 |
| Body mass index, n (%) |  |  |  |
| Underweight (<18.5 kg/m^2^) | 0 | 1 (3.3) | 0 |
| Normal weight (≥18.5–24.9 kg/m^2^) | 13 (43.3) | 10 (33.3) | 7 (23.3) |
| Overweight (≥25.0–29.9 kg/m^2^) | 9 (30.0) | 10 (33.3) | 10 (33.3) |
| Obese (≥30.0 kg/m^2^) | 8 (26.7) | 9 (30.0) | 13 (43.3) |

Abbreviations: N-binding, SARS-CoV-2 nucleoprotein‒binding; NAAT, nucleic acid amplification test.

^a^Includes multiracial.

^b^Positive N-binding antibody result at baseline, positive NAAT result at baseline, or medical history of COVID-19.

^c^Negative N-binding antibody result at baseline, negative NAAT result at baseline, and no medical history of COVID-19.

## Table S5. Summary of AEs Through 6 Months After Study Vaccination in the Sentinel Cohort (Safety Population)

| **Adverse Event** | **Bivalent BA.1 60 µg (n=30)** | **Bivalent BA.1 30 µg (n=30)** | **Monovalent BA.1 60 µg (n=30)** |
| --- | --- | --- | --- |
| Any AE, n (%) | 4 (13.3) | 5 (16.7) | 6 (20.0) |
| Related | 3 (10.0) | 2 (6.7) | 4 (13.3) |
| Any SAE, n (%) | 0 | 0 | 0 |
| Any nonserious AE, n (%) | 4 (13.3) | 5 (16.7) | 6 (20.0) |
| Related | 3 (10.0) | 2 (6.7) | 4 (13.3) |
| Any AE leading to withdrawal, n (%) | 0 | 0 | 0 |
| Death, n (%) | 0 | 0 | 0 |

Abbreviations: AE, adverse event; SAE, serious adverse event.

## References

1. Food and Drug Administration. Development and licensure of vaccines to prevent COVID-19. Guidance for industry. Available from: https://www.fda.gov/media/139638/download. Accessed: September 2, 2025.
2. Centers for Disease Control and Prevention. People with certain medical conditions. Available from: https://www.cdc.gov/covid/hcp/clinical-care/underlying-conditions.html. Accessed: September 2, 2025.
